# How to Reduce Excessive Use of the Health Care Service in Medical Aid Beneficiaries: Effectiveness of Community-Based Case Management

**DOI:** 10.3390/ijerph17072503

**Published:** 2020-04-06

**Authors:** Myung Ja Kim, Eunhee Lee

**Affiliations:** 1 Department of Resident Welfare, Hwacheon County office, Hwacheon-gun, Ganwon-do 24125, Korea; hwab4018@korea.kr; 2 School of Nursing/Research Institute of Nursing Science, Hallym University, Chuncheon, Gangwon-do 24252, Korea

**Keywords:** case management, medical aid program, health care utilization, health care cost

## Abstract

Community-based case management for medical aid beneficiaries was implemented in Korea to promote the rational use of medical care and stabilize the financial system. This study investigated the economic impact of community-based case management on reductions in healthcare utilization and costs. This was a program study using a national database to evaluate the effectiveness of community-based case management in changing not only healthcare utilization and costs but also client-centered outcomes using the NHI database and 198 regional databases. A total of 1741 case management clients were included in this study. The case management clients were categorized into three targeted groups and were provided individualized services according to the groups. Client-centered outcomes, such as health-related quality of life (QOL), self-care ability, and having a support system, increased after case management. Healthcare utilization and costs decreased significantly after case management. However, there was no significant difference in the decrease between the groups. An increase in healthcare utilization among medical aid beneficiaries has been observed due to the aging population and an increase in the number of recipients. To reduce healthcare utilization and costs while maintaining the health status of the beneficiaries, it is necessary to expand targeted case management.

## 1. Introduction

The entire national population in Korea is covered by a healthcare system namely, the National Health Insurance (NHI) program or Medical Aid Program. Most Koreans (96%) are covered by the NHI program, while 3–4% of the people who are unable to pay for their own healthcare coverage are covered by the Medical Aid Program [1,2]. Hence, the Medical Aid Program is considered a social security system, which covers medical services, such as illness, injuries, and childbirth, to prevent health effects caused by poverty [3,4,5]. The Medical Aid Program is financed by both the central government and the local government and is classified as type 1 or type 2 based on whether someone is incapable (those under 18 or over 65 years of age or disabled) or capable of working [2,6]. Type 1 has no copayment for any medical use, whereas type 2 includes some deductions. However, even the out-of-pocket payments (OOPs) amount for type 2 is much lower compared to that for people covered by the NHI program [6].

Since most of the recipients covered by the Medical Aid Program are medically underserved populations, such as the elderly, disabled, or those ill with a rare disease [7,8], they might use medical services frequently. However, even considering the vulnerability of the beneficiaries, healthcare utilization and costs for these populations increased dramatically [6]. Additionally, healthcare utilization and costs for medical aid beneficiaries are much higher than for others covered by the NHI [6,9]. This circumstance might be partly attributable to the increase in medical fees, which have increased 1–3% annually [10]. However, this might be caused by the Medical Aid Program insurance system, which has little or no OOPs for Medical Aid Program. Previous studies reported that the low perception of medical costs by recipients and oversupply might lead to an increase in the healthcare medical aid costs [7,9].

To promote the rational use of medical care by beneficiaries and stabilize the financial system, community-based case management for medical aid beneficiaries was initiated in 2003 and has been implemented based on local government in order to manage the medical use behavior of the beneficiaries who use various medical institutions. Community-based case management was first targeted to the beneficiaries who used an excessive amount of outpatient services [2,5]. Subsequently, community-based case management was extended to long-term inpatients, and lastly to the beneficiaries who used outpatient and inpatient services repeatedly [5]. Currently, community-based case management is categorized into three groups according to the medical utilization pattern that provides customized services for each need. Once the medical aid beneficiaries were enrolled, case managers in each community assessed the health status and medical use of all beneficiaries in the community, primarily to determine whether case management was needed or not. Next, considering the pattern of healthcare services utilization, the beneficiaries eligible for case management were classified into three target groups: (1) the excessive use of outpatient service, (2) long-term inpatients, and (3) the repeated use of inpatient and outpatient services. Case managers provided individualized services suitable for each group. It has established as a management system that compensates for the potential problems that might occur within the Medical Aid Program to protect the vulnerable population.

Several studies reported that case management for medical aid beneficiaries was effective in reducing healthcare use [11,12,13,14]. However, most of these studies focused on reducing healthcare use to demonstrate the effectiveness of case management. Hence, there is a lack of research on other effectiveness outcomes, such as changes in health status and the capability to use healthcare services reasonably. However, if these outcomes are not increased remarkably, healthcare utilization and cost might increase again after proving case management. Therefore, case management should induce the improvement of health status and the capability of the rational use of healthcare services, and the improvement of these outcomes needs to be analyzed as the effectiveness of case management. Additionally, despite providing differentiated services for each group, there was a lack of differential analyses in the effectiveness of case management concerning each group. Therefore, this study aimed to analyze the effectiveness of case management on reducing healthcare utilization and cost and changing health status and capability about healthcare use in the three groups.

## 2. Materials and Methods

This study used databases from the NHI and 198 regions to evaluate the effectiveness of case management on changes in client-centered outcomes and excessive healthcare utilization and costs. In databases, the identification of the beneficiary and case manager are all stored in an encrypted format for protecting personal information. This study has been approved by the Ethics Committee of Hallym University (No. 2017-06-29). Informed consent to each participant was exempted by the board.

### 2.1. Sample and Data Source

This study included medical beneficiaries who enrolled in the case management in 2016. We only included the subjects who had enrolled and terminated case management in 2016 and excluded the subjects who terminated early due to transfer or death. The case manager, a registered nurse or social worker, provided an initial assessment of all the medical beneficiaries regarding the need for case management and divided the clients into three different target groups. The three target groups were the outpatient target group associated with excessive use of outpatient services, the inpatient target group of long-term inpatients, and the repeated use target group of beneficiaries who repeatedly used inpatient and outpatient services according to healthcare utilization patterns. The criteria for selection of case manager clients and classification into each group follows the guidelines [15] and applies the same criteria in all regions. The outpatient target group was defined as beneficiaries who frequently used healthcare services for the same disease. The inpatient target group was defined as beneficiaries who had been hospitalized for more than a month. The repeated use target group was defined as beneficiaries from the top 30% of healthcare users among all beneficiaries enrolled in case management.

Among the case management clients, approximately 60% is the outpatient target, 25% is the inpatient target, and 15% is the repeated use target. Moreover, there exists a specific pattern of healthcare utilization based on regions in Korea, such as urban and rural. Therefore, this study employed stratified proportion sampling reflecting the entire proportion of the three target groups and regions. Consequently, 1741 clients, consisting of 1107, 351, and 283 clients from the outpatient target group, the inpatient target group, and the repeated use target group were included in the present study.

The data on healthcare utilization and costs were extracted from the NHI database in Korea. In the case of health assessment data submitted by the case managers, after approval, we extracted the clients’ health assessment data from a database of 198 regions. After extracting the data from the two databases, we built an analytic dataset by assembling the health assessment data from the regional database, healthcare utilization, and costs data from the NHI database.

### 2.2. Variables

The data collected from the database of 198 regions included general characteristics of the clients, services performed by the case managers, and assessment data. The general characteristics were gender, age, medical aid type, re-enrollment, residence status, the presence of a housemate, education level, region, and main disease. Case managers provided services by home visit, phone, mail, hospital visit, or transitional care by acting as a resource liaison between the hospital, other facilities, and home, and referral to a hospital. We calculated the total amount of services and frequency of each service method per person. Additionally, the data extracted from the NHI database included healthcare costs and healthcare utilization such as inpatient and outpatient service.

The primary outcome of case management was the change in the client-centered outcome such as health-related quality of life (QOL), self-care ability, and having a support system, which was analyzed using health assessment data. Health-related QOL was measured by a short questionnaire for the outpatient target group and inpatient target group and Euro-quality of life-5 dimension (EQ-5D) for the repeated use target group. A short questionnaire for measuring health-related QOL was developed by the Ministry of Health and Welfare, which consisted of six items each with a 5-point Likert scale. The repeated use target group categorized for case management used the latest more valid instrument, the EQ-5D, for measuring health-related QOL accurately. Self-care ability and a having support system were also measured using a questionnaire developed by the Ministry of Health and Welfare, which were investigated only in the outpatient target group and the inpatient target group. The questionnaire of self-care ability consists of six items, each with a 5-point Likert scale. All items of self-care were investigated in the outpatient target group, whereas five items, excluding one irrelevant question, were investigated in the inpatient target group. Having a support system was measured using two items on a 5-point Likert scale.

Secondary outcomes were defined as changes in actual healthcare utilization and costs. Healthcare utilization referred to inpatient days and outpatient visits. Healthcare costs included only medical costs associated with healthcare, such as treatment costs, hospitalization costs, and drug costs, and did not estimate non-medical costs and indirect costs such a productivity loss. The medical cost was extracted from the NHI database and estimated using a bottom-up approach. We extracted the data of healthcare utilization and costs for the year (2015) before the initiation of case management and for the year (2016) after the service.

### 2.3. Analysis

To summarize the data, the general characteristics of the case management clients are reported as the mean and standard deviation for continuous variables and frequencies and percentages for categorical variables. We used a Chi-squared test and ANOVA to determine whether the general characteristics were significantly different between the target groups. Service performance was reported as total frequency and the average number of services per person. Changes in client-centered outcomes and healthcare utilization and costs between before and after case management were analyzed by paired t-tests. Lastly, the differences in healthcare utilization and costs between the target groups were analyzed by ANCOVA by adjusting for the variable that showed differences based on the groups. The collected data were analyzed using SPSS WIN 22.0 (Chicago, IL, USA) and Stata SE version 14 (StataCorp LP., College Station, TX, USA).

## 3. Results

### 3.1. Differences in General Characteristics of the Target Groups for Case Management

Table 1 shows the general characteristics of the clients enrolled in case management and differences in the general characteristics of the target groups. The target group for case management was divided into three groups, outpatient target, inpatient target, and repeated use target. The outpatient target group and the repeated use target group had more than 60% women, whereas the proportion of women in the inpatient target group was lower than in the other groups (X^2^ = 25.039, *p* < 0.001). The proportion of over 65 years was significantly higher in the outpatient target group (70.8%, X^2^ = 12.950, *p* = 0.002). Approximately 95% of the clients were covered by the type 1 medical aid program, and a similar pattern was observed in all groups. Typically, more than 60% of the clients received case management more than twice, which was different between the groups (X^2^ = 28,766, *p* < 0.001). Of the clients, the proportion who lived alone was higher than that of having a housemate in all groups, and the proportion was highest in the inpatient target. More than 60% of the clients lived in small cities or rural areas and approximately 70% of the clients had an elementary school education or less. The most frequent disease was circulatory diseases, which showed a similar distribution in the outpatient target and repeated use target groups. The proportion of mental disorders, such as dementia, accounted for 10–20% in both the outpatient target and repeated use target groups, whereas it was above 30% in the inpatient target group.

### 3.2. Service Duration and Performance of Case Management by Target Groups

The case management service duration and performance status are shown in Table 2. The duration of the service provided was up to 12 months in the repeated use target group, which was longer compared to the other groups. As short-term management for this group could not induce enough change in healthcare behavior, from the beginning, case management for the repeated use target group was designed to provide 12 months of case management. The outpatient target group was provided case management services for a short duration of three months. However, this group was provided with more services per month. The average number of services used per person in the repeated use target group was 16.20, which was the highest of all the groups. However, since they were provided services for 12 months, the monthly average was not the highest.

Case management services were delivered by telephone, mail, home visit, transitional care (resource liaison), hospital visit, and referral to a hospital. The service method showed a similar distribution in all the target groups. The phone call was the most commonly used service among the entire services for every group, followed by mail, home visits, and transitional care. The frequency of hospital visits and referrals to other institutions was low in all the groups. Considering that the service duration was different for the target group, the outpatient target group received an enormous amount of service within a short period of time.

### 3.3. Changes in Service Outcomes for Case Management

The health-related QOL in the outpatient target group before enrollment in case management was 17.20, which was lower than that in the inpatient target group (18.53) (Table 3). After providing case management, the health-related QOL was significantly improved in all of the groups. Compared to the long-term inpatient group, the health-related QOL was significantly improved in the outpatient target group (*t* = 29.290, *p* < 0.001). Not only health-related QOL but also other client-centered outcomes such as self-care ability and having a support system increased significantly after case management.

**Figure 1:**
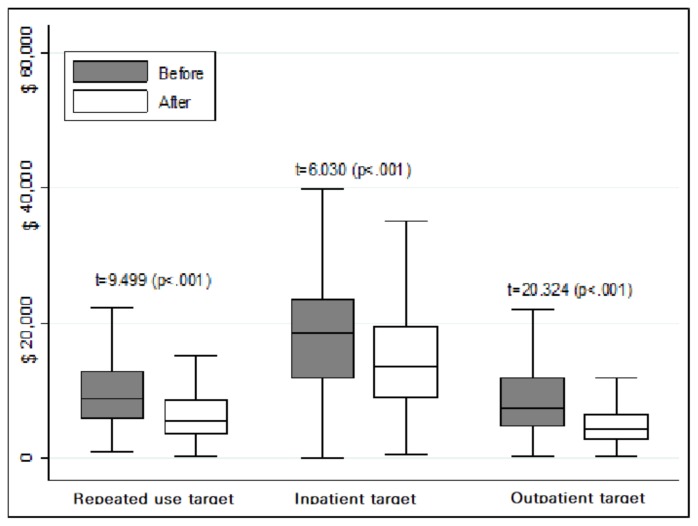
Changes in health care cost in the targeted group.

Before enrollment for case management, the inpatient days in a year were estimated as 240.2 days in the long-term inpatient group, which was extremely high compared to the other groups. The outpatient visits in a year were relatively high in the outpatient target and repeated use target groups. Healthcare utilization in both the inpatients and outpatients showed a statistically significant decrease after case management in all groups (Table 3). Moreover, healthcare costs showed a significant decrease after case management in all the groups, as well (Figure 1).

### 3.4. Comparison of Effect on Case Management by the Target Groups

Differences in healthcare utilization and healthcare cost by the groups were analyzed by adjusting the variables and the differences were statistically significant (Table 4). The inpatient days changed a significant difference among the target groups. Inpatient days were mainly reduced in the inpatient target group, whereas outpatient visits were reduced mainly in the repeated use target group. A great decrease in healthcare cost per client was observed in the outpatient target group, followed by the inpatient target group and repeated use target group. However, there was no significant difference between the different groups.

## 4. Discussion

Community-based case management showed positive effects not only in changing client-focused outcomes, such as health-related QOL and the perception of rational medical use but also in reducing healthcare utilization and costs, consistent with the results of previous studies [11,12,13,14]. The excessive use of healthcare by medical aid beneficiaries is not confined to Korea. In the United States and Japan, comprehensive health insurance coverage with zero cost-sharing has led to an increase healthcare spending [4,16,17]. To reduce healthcare utilization and costs for medical aid beneficiaries, many countries have introduced case management and demonstrated an effect on reducing healthcare use and expenditure [11,12,18]. Case management in Korea also showed a positive effect in reducing healthcare utilization in this study.

A major decrease in total hospital visit days was seen in the repeated use target group, followed by the long-term inpatient group, and the outpatient target group. However, no significant difference was observed among the groups by adjusting for preexisting utilization. The repeated use target group was provided service for the longest period and the decrease in utilization seen in this group was similar to the other groups. Hence, the effectiveness of case management in the repeated use target group was probably not greater than that in the other groups. This result could be attributed to the characteristics of the repeated use target group. As the repeated use target group had consumed an extremely large amount of healthcare services among all beneficiaries enrolled in case management, the short-term management provided to this group could not induce enough change in healthcare behavior. Therefore, the Korean government set up a suitable service model for the repeated use target group. Hence, community-based case management in Korea is a systematic model in the aspect of categorizing target groups and providing appropriate services to each group to achieve similar levels of effectiveness.

The healthcare costs also decreased significantly in all the groups after case management. However, after adjusting for the baseline costs, no significant differences in reduction were observed among the groups. Despite providing a shorter service period to the outpatient target group, healthcare costs were reduced the most in this group among all of the groups. Hence, in terms of efficiency, case management showed the best performance in the outpatient target group. The inpatient target group had a longer service period than the outpatient target group, while it did not show more effective results. Unlike other groups, the inpatient target group showed a significantly lower rate (26.8%) of housemates compared to the other groups (39.6–42.5%). Beneficiaries without housemates are not provided caring service at home if they become ill. Thus, patients living alone are more likely to stay in the hospital longer when hospitalized [1,7,19]. In this study, the inpatient target group had the option to totally rely on inpatient care if they were unable to obtain appropriate care as an outpatient. Under this circumstance, case management for the inpatient target group could not be an effective way to reduce healthcare utilization and costs compared to other groups. Since support systems should be in place in advance of patient discharge, the effect of case management through resource liaison will be maximized.

The client-centered outcome such as health-related QOL and perception of self-care could not be directly compared because the measurement instruments were different. Health-related QOL was measured by different instruments in the groups, and the perception of self-care was not investigated in the repeated use target group. Among the client-centered outcomes, health-related QOL improved after the case management in all groups significantly, which is consistent with other studies [13,20,21]. Moreover, improvements in the client-centered outcomes led to reductions in healthcare utilization and costs. Additionally, the self-care ability must be increased to maintain the rational use of healthcare services, even after case management ends. Therefore, primary outcomes should be measured periodically using valid instruments for evaluating the outcomes of case management.

The majority of the clients for community-based case management were elderly. As the medical aid program in Korea targets vulnerable groups, the proportion of the elderly enrolled as beneficiaries for case management is expected to increase. The aging of case management clients might cause an increase in healthcare utilization and costs, which is the same phenomenon in the NHI [22,23,24]. Thus, the need for a management system will continue to be addressed for managing healthcare utilization and costs.

In this study, most of the clients were covered by type 1 medical aid program. The extremely low OOPs required of type 1 medical aid recipients might lead to increased healthcare utilization and costs. Previous studies reported that the more the patients were covered by insurance, the more they used medical services [1,2,4]. Hence, insurance type or insurance coverage affect healthcare utilization. Notwithstanding, health policies, such as the medical aid program, should be maintained as they are a social safety net for the protection of vulnerable populations. However, at the same time, it needs to monitor and manage whether beneficiaries use healthcare services rationally and appropriately. That is the reason the Korean government implemented community-based case management.

As previously mentioned, low OOP might lead to the excessive use of healthcare services. However, individual health status is the basic, critical factor related to healthcare utilization. Additionally, health status and self-care ability are deeply influenced by social factors, such as economic status and education level [1,14]. Specifically, the education level is highly related to health literacy and contributes to an individual’s health [25]. In this study, the majority of clients had an education level of only elementary school or less. Hence, it is difficult to recognize their health status objectively and use proper resources for maintaining their health. Thus, improving health literacy through case management is needed.

In this study, the majority of clients were re-enrolled, which means that most of the clients still needed a large amount of medical services due to disease, or an increase in repeat medical use after the termination of service. This suggests that the sustainability of case management declined with time. Additionally, service duration might affect re-enrollment. The outpatient target group was given the shortest period of services, but re-enrollment was the highest in this group. Not only the outpatient target group but also the other groups were provided services for quite a short time compared to other countries [12]. As most of the clients for case management had several diseases and they depended on healthcare services, the pattern of healthcare utilization was difficult to change in a short time. It is essential to provide the right amount of services during the right period for achieving the desired effect. Further research is required to evaluate the service period for each group.

## 5. Conclusions

This study investigated the economic impact of case management on healthcare utilization and costs. Health-related QOL and self-care ability were increased significantly, and healthcare utilization and costs were reduced significantly after case management. Despite the different characteristics of each group, the reduction in healthcare utilization and costs by targeted management were similar.

## Figures and Tables

**Table 1:** General characteristics of clients enrolled in community-based case management.

Variables	Categories	Target Group (*n* = 1741)			X^2^	*p*-Value
		Outpatient (*n* = 1107)	Inpatient (*n* = 351)	Repeated Use (*n* = 283)		
Gender	Women	729(65.9)	179(51.0)	175(61.8)	25.039	<0.001
	Men	378(34.1)	172(49.0)	108(38.2)		
Age	Under 64 years	323(29.2)	133(37.9)	105(37.1)	12.950	0.002
	Over 65 years	784(70.8)	218(62.1)	179(62.9)		
Medical care type	Type1	1052(95.0)	343(97.7)	272(96.1)	4.845	0.089
	Type2	55(5.0)	8(2.3)	11(3.9)		
Enrollment	Re-enroll	842(76.1)	216(61.5)	198(70.0)	28.766	<0.001
	First enroll	265(23.9)	135(28.5)	85(30.0)		
Housemate	No	637(57.5)	257(73.2)	171(60.4)	27.653	<0.001
	Yes	470(42.5)	94(26.8)	112(39.6)		
Education	Pre-primary	485(43.9)	135(38.5)	110(28.9)	5.530	0.478
	Primary	305(27.6)	99(28.2)	84(29.7)		
	Lower secondary	143(12.9)	49(14.0)	40(14.1)		
	Upper secondary	174(15.6)	68(19.3)	49(17.3)		
Region	Metropolitan	381(34.4)	127(36.2)	107(37.8)	2.358	0.670
	Non-metropolitan	375(33.9)	121(34.5)	98(34.6)		
	Rural	351(31.7)	103(29.3)	78(27.6)		
Main disease	Cardiovascular	394(35.6)	107(30.5)	85(30.0)	121.952	<0.001
	Musculoskeletal	209(18.9)	35(10.0)	72(25.5)		
	Mental disorder	137(12.4)	113(32.1)	39(13.8)		
	Endocrine disease	183(16.5)	21(6.0)	42(14.8)		
	Neoplasm	104(9.4)	55(15.7)	27(9.5)		
	Others	80(7.2)	20(5.7)	18(6.4)		

**Table 2:** Service duration and performance of community-based case management by target groups.

Variables	Categories	Outpatient Target	Inpatient Target	Repeated Use Target
		(*n* = 1107)	(*n* = 351)	(*n* = 283)
Duration (months)		3	5	within 12
Frequency of service, n(%)	Total	9814	3651	4584
	Phone	4814(49.1)	1946(53.3)	2292(50.0)
	Mail	2503(25.5)	784(21.5)	1078(23.5)
	Home visit	2278(23.2)	783(21.4)	945(20.6)
	Transitional care	160(1.6)	115(3.1)	176(3.8)
	Hospital visit	50(0.5)	3(0.1)	82(1.8)
	Referral to hospital	9(0.1)	20(0.5)	11(0.2)
Number of services per person, mean ± SD	Total	8.87 ± 1.54	10.40 ± 4.10	16.20 ± 6.00
	Phone	4.35 ± 0.78	5.54 ± 2.58	8.10 ± 3.27
	Mail	2.26 ± 1.02	2.23 ± 1.42	3.81 ± 2.53
	Home visit	2.06 ± 0.36	2.23 ± 1.11	3.34 ± 1.49
	Transitional care	0.14 ± 0.43	0.33 ± 0.61	0.62 ± 1.09
	Hospital visit	0.05 ± 0.24	0.01 ± 0.10	0.29 ± 1.14
	Referral to hospital	0.01 ± 0.10	0.06 ± 0.23	0.04 ± 0.21

**Table 3:** Service outcome of community-based case management by the target group.

Variables	Group	Before	After	*t*	*p*-Value
		(Mean ± SD)	(Mean ± SD)		
Client-centered outcome					
Health related QOL	Outpatient target	17.20 ± 2.98	19.33 ± 2.38	29.29	<0.001
	Inpatient target	18.53 ± 4.00	19.45 ± 4.14	8.675	<0.001
	Repeated use target *	0.68 ± 0.14	0.78 ± 0.13	11.912	<0.001
Self-care ability	Outpatient target	15.41 ± 2.82	18.64 ± 2.52	37.152	<0.001
	Inpatient target	12.64 ± 4.12	14.05 ± 4.49	10.507	<0.001
Having support system	Outpatient target	5.85 ± 1.70	6.43 ± 1.45	17.054	<0.001
	Inpatient target	6.32 ± 1.73	6.99 ± 1.65	9.842	<0.001
Health care utilization					
Inpatient days	Outpatient target	30.5 ± 23.0	10.6 ± 30.7	17.766	<0.001
	Inpatient target	240.2 ± 141.3	206.0 ± 117.6	3.924	<0.001
	Repeated use target	36.1 ± 64.1	18.7 ± 49.2	5.898	<0.001
Outpatient visits	Outpatient target	128.3 ± 120.4	104.7 ± 111.1	8.716	<0.001
	Inpatient target	47.3 ± 93.9	34.5 ± 56.1	2.540	0.012
	Repeated use target	182.6 ± 137.1	138.9 ± 119.6	9.821	<0.001

* Note: Distribution of health-related QOL for the repeated use target group was different from other groups because the instrument of health-related QOL for the repeated use target group was different.

**Table 4:** Comparison of changes in health care utilization and cost per client by target groups.

Variables	Outpatient Target ^a^	Inpatient Target ^b^	Repeated Use Target ^c^	F	*p*-Value	Post-hoc
	(*n* = 1107)	(*n* = 351)	(*n* = 283)			
Healthcare utilization						
Inpatient days	19.9 ± 44.6	34.3 ± 163.3	17.4 ± 49.2	4.45	0.012	b > a,c
Outpatient visits	23.3 ± 90.2	12.9 ± 93.9	43.1 ± 74.5	9.73	0.000	c > a,b
Healthcare cost, $	4007.9 ± 6519.6	3592.6 ± 11,002.9	3197.0 ± 5621.9	1.46	0.233	
